# Field-ridge structured bioactive Zn-based scaffold promotes bone regeneration via cellular mechanotransduction

**DOI:** 10.1016/j.bioactmat.2026.07.048

**Published:** 2026-08-05

**Authors:** Xuanhe Feng, Dongxu Xie, Qiunan Zhou, Wanzhen Lei, Yilin He, Chen Zhang, Siyuan Ouyang, Lei Hu, Peng Wen, Luyuan Jin

**Affiliations:** a Emergent Dental Care and General Dentistry, Beijing Stomatological Hospital, Capital Medical University, Beijing, 100070, China; b State Key Laboratory of Clean and Efficient Turbomachinery Power Equipment, Tsinghua University, Beijing, 100084, China; c Department of Mechanical Engineering, Tsinghua University, Beijing, 100084, China; d Beijing Institute of Dental Research, Beijing Stomatological Hospital, Capital Medical University, Beijing, 100070, China; e Department of Oral Implantology, Peking University School and Hospital of Stomatology, Beijing, 100871, China; f Department of Prosthodontics, Beijing Stomatological Hospital, Capital Medical University, Beijing, 100070, China

**Keywords:** Zn-Li alloy, Laser powder bed fusion, Porous scaffold, Surface texture, Mechanotransduction, Bone regeneration

## Abstract

Large bone defects often exhibit impaired healing due to the absence of a favorable osteoinductive microenvironment at the defect core. Drawing inspiration from agricultural field-ridge structures, we developed a Zn-based metallic biomimetic scaffold, designated the “Osteogenic Conditioning Scaffold” (OCS), with a tailored porous architecture and groove-ridge-like surface texture to enhance bone conduction and osteogenesis. Using a rationally designed laser scanning strategy in laser powder bed fusion (L-PBF), we spatially regulated melt-track overlap to generate ordered groove-ridge-like textures, enabling the one-step fabrication of 3D-printed Zn-0.4Li porous scaffolds with oriented surface textures. *In vitro* evaluations revealed that the OCS promoted the adhesion and spreading of bone marrow mesenchymal stem cells (BMSCs), activated mechanotransduction signaling (upregulated VCL, phosphorylated FAK, and nuclear translocation of YAP), and triggered epigenetic regulator shifts (downregulated KDM5A and upregulated KDM6A), thereby enhancing osteogenic differentiation. In a rabbit critical-sized calvarial defect model, the scaffold significantly accelerated new bone formation and osseointegration, with consistent mechanistic signatures validated *in vivo*. This robust, stable, and readily tunable fabrication approach enables the synergistic integration of osteogenic bioactivity from Zn^2+^ and Li^+^ ions with the osteoinductive effects of hierarchical porous and groove-ridge-like textures, offering a promising strategy for the repair of large bone defects.

## Highlights

• In situ L-PBF produces oriented groove-ridge textures on inner pore walls.
• One-step L-PBF fabricates Zn-0.4Li TPMS Osteogenic Conditioning Scaffold (OCS).
• OCS optimizes cytocompatibility and osteogenic activity for bone repair.
• OCS modulates mechanotransduction and histone demethylases.

## Graphical abstract

**Figure:**
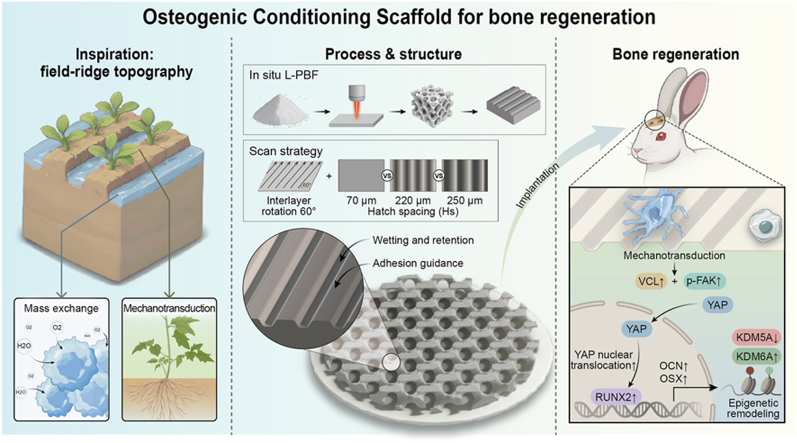

## 1 Introduction

Large bone defects caused by trauma, infection, tumors, or congenital disorders remain a global clinical challenge [1]. In the hostile defect microenvironment, insufficient recruitment and osteogenic differentiation of endogenous stem cells, together with the limited ability of scaffolds to actively regulate cell behaviors [2,3], are key reasons for suboptimal bone regeneration in tissue engineering [4,5]. Such limitations are particularly pronounced in the defect core, where poor vascular supply and restricted mass transport hinder stable cell colonization within pores, thereby constraining osteogenic initiation and sustained regeneration. Current approaches often rely on exogenous growth factors (e.g., BMP-2/VEGF) or cell-based therapies (e.g., transplantation of BMSCs/MSCs and delivery of their exosomes) to enhance osteogenesis and angiogenesis, yet they are associated with high cost, difficulties in controlled delivery, and translational risks such as immune reactions and ectopic bone formation [6,7]. Therefore, developing robust strategies that strengthen scaffold-mediated regulation of endogenous cell adhesion, engraftment, and osteogenic commitment remains an urgent need.

In bone tissue engineering, scaffolds are expected not only to fill defects but also to provide a microenvironment that supports endogenous cell adhesion, infiltration, and osteogenic commitment. Inspired by the groove-ridge topography generated by agricultural tillage, we hypothesized that oriented groove-ridge-like textures could improve local fluid retention, provide anchoring tracks, and guide cell organization within porous scaffolds. Analogously, oriented stripes or groove-ridge-like textures on material outer surfaces have been widely shown to guide cell behaviors [8]. For instance, on parallel microgrooved titanium, cells elongate and align along the grooves, accompanied by oriented stress fiber formation and focal adhesion maturation, which enhances migration and adhesion stability [9]. Osteon-mimetic concentric microgrooves can similarly direct BMSC alignment and improve osteogenesis-related phenotypes, potentially involving epigenetic regulation linked to histone modifications [10].

Beyond structural guidance, the scaffold material itself should also provide a bioactive microenvironment. Biodegradable Zn-Li alloys, owing to their favorable biocompatibility and intrinsic pro-osteogenic potential, have been widely considered promising candidates for bone repair [11,12]. Recent Zn-based systems have further shown that alloy or composite design can integrate mechanical reinforcement, controllable degradation, and additional biological functions, including antibacterial, antitumor, and osteogenesis-related properties [[13], [14], [15]]. Among these materials, Zn-Li alloys are particularly attractive because released Zn^2+^ and Li^+^ can participate in transcriptional regulation and mineralization processes relevant to osteogenesis [16], providing a bioactive basis for the defect site. However, in large-volume bone defects, the most critical bottleneck is often inside the scaffold, especially in the defect core where cell infiltration and mass exchange are limited. This creates a strong rationale for introducing field-ridge-inspired oriented microstructures into Zn-Li porous scaffolds, particularly on the inner pore-wall surfaces.

Nevertheless, fabricating orientation-controlled groove-ridge-like textures on the inner surfaces of customized 3D porous scaffolds remains challenging. Existing approaches for oriented groove-ridge-like textures typically rely on laser texturing, chemical etching, or imprinting, which are generally suited to planar or cylindrical substrates [17]. For porous architectures with continuous curvature and pronounced pore occlusion, the inner pore walls are difficult to access uniformly, making it hard to reproduce orientation-consistent textures throughout the scaffold. In practice, sandblasting and acid etching are more commonly applied to porous scaffolds to increase roughness [18], yet the resulting microstructures are largely random and provide limited, inconsistent directional cues [19,20]. Meanwhile, clinical translation increasingly demands patient-specific porous designs that match defect anatomy, further amplifying the need for a manufacturable strategy that enables controllable groove-ridge-like textures on the inner surfaces of customized porous scaffolds.

L-PBF fabricates customized metallic porous scaffolds via computer-controlled laser scanning and melting-solidification. By regulating laser energy input and scanning paths, fine micropores can be *in situ* generated on melt tracks upon solidification. In our previous work, porous scaffolds with hierarchical pores were produced by tuning laser energy and hatch spacing, which exhibited high specific surface area and groove-ridge surface morphology [21]. This technique not only creates porous architectures matching the anatomical geometry of bone defects, but also modulates the spatial distribution of internal micropore stacking by adjusting interlayer rotation and hatch spacing, thereby enabling controllable formation of surface groove-ridge-like textures without post processing. This approach allows the simultaneous integration of customized porous geometry and oriented groove-ridge-like textures within a single reliable manufacturing workflow.

**Fig. 1:**
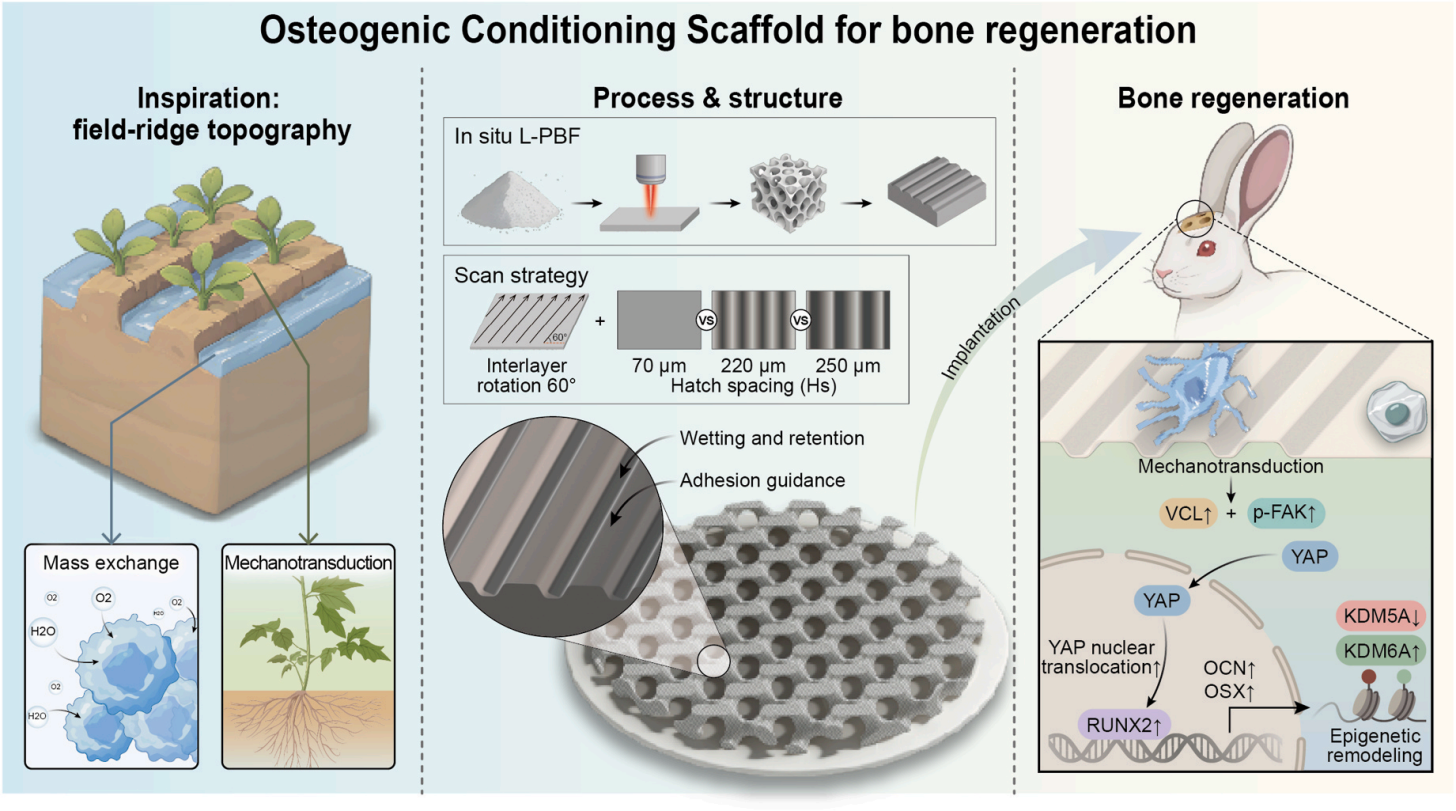
Schematic diagram of Osteogenic Conditioning Scaffold (OCS) for bone regeneration.

Here, we developed an Osteogenic Conditioning Scaffold (OCS) that couples a bioactive Zn-Li platform with field-ridge-inspired groove-ridge-like textures to condition the defect-core microenvironment (Fig. 1). By optimizing digital design and L-PBF parameters, we constructed regularly oriented groove-ridge-like textures *in situ* on the inner pore-wall surfaces of Zn-0.4Li customized scaffolds. This “material-structure-function” integrated design allows the defect core to benefit from two complementary “functional soil” roles: the Zn-Li active metal platform provides a bioactive microenvironmental basis, while the oriented groove-ridge-like textures contribute directional interfacial cues that support cell adhesion and mechanotransduction. The resulting OCS design facilitates rapid wetting and transient fluid retention to support local mass exchange, while the continuous ridges provide potential anchoring tracks and directional contact-guidance cues. We further show that this integrated interface exerts a pronounced conditioning effect on BMSCs, converting mechanical cues into adhesion-related responses and mechanotransduction, while being associated with enhanced osteogenic differentiation and shifts in osteogenesis-related epigenetic regulators. Importantly, these effects are not transient. In a rabbit critical-sized calvarial defect model, the scaffold retained enhanced osteogenic performance during the early healing phase, indicating that the integrated interfacial cues of the OCS remained functionally relevant even as the scaffold gradually degrades. Together with bioactive metal species, this material-structure integrated design establishes a more instructive “functional soil” microenvironment in the defect core, providing sustained and controllable support for endogenous cell adhesion, engraftment, and osteogenic differentiation to promote bone regeneration.

## 2 Materials and methods

### 2.1 Preparation of Zn-Li alloy TPMS scaffolds

Triply periodic minimal surface (TPMS, Gyroid type) scaffolds were designed using Topology software with a unit cell size of 1.5 mm and target porosity of approximately 65%. Scaffolds were fabricated using a commercial L-PBF (BLT, China), equipped with a single-mode ytterbium fiber laser (IPG YLR500, Germany), with a focal diameter of 70 μm and a working wavelength of 1070 nm. Zn-0.4Li alloy spherical powder, within 15-45 μm diameter, was used. During processing, the chamber was filled with argon gas, maintaining a purity of over 99.99% and keeping the oxygen content below 120 ppm during the melting process. A pure Zn plate was used as the substrate, which was ground and cleaned with ethanol prior to each printing session [21].

The key parameters of the L-PBF process include laser power (P), scanning speed (Vs), hatch spacing (Hs), and layer thickness (Ds). Through optimization of the processing parameters, Ds was set to 20 μm, while P and Vs were set to 40 W and 800 mm/s, respectively. The values of Hs were established at 70 μm, 100 μm, 220 μm, 250 μm, and 280 μm. The internal scanning employed a zigzag pattern, with a 67° rotation for each layer when Hs was set to 70 μm, and a 60° rotation for layers with Hs values ranging from 100 to 280 μm. Biomimetic TPMS structures were fabricated in various sizes for different tests: ϕ10 × 2 mm for *in vitro* cell and electrochemical testing, and 6 × 6 × 6 mm for mechanical and dynamic immersion tests. The dimensional accuracy of the printed scaffolds was evaluated by measuring representative structural features using high-resolution optical microscopy and SEM. Based on these measurements, the achievable feature-level dimensional fidelity under the optimized parameters was on the order of 20 μm. Notably, the shrinkage behavior of the melt pool during laser processing has been fully considered during parameter optimization, including adjustments to laser power, scanning speed, and hatch spacing. As a result, no significant macroscopic shrinkage or dimensional distortion was observed after printing.

To regulate the melt pool overlap and defect stacking, layer-to-layer rotation angles of 0°, 60°, and 67° were applied. Based on mechanical anisotropy analysis, the 60° rotation strategy was selected for subsequent biological experiments. Groove-ridge-like textures were formed intrinsically through controlled stacking of micropore boundaries. Three independent printing batches were fabricated to ensure reproducibility. After fabrication, samples were ultrasonically cleaned in absolute ethanol and dried under vacuum.

### 2.2 Microstructure and mechanical characterization

The surface microarchitecture of the samples was examined using scanning electron microscopy (SEM, Gemini 300, Zeiss, Germany) equipped with an energy-dispersive X-ray spectroscopy system (EDS, X-Max 20, Oxford Instruments, UK) for elemental analysis. Three-dimensional surface morphology was further reconstructed using confocal laser scanning microscopy (CLSM, LSM900, Zeiss, Germany). Interior porosity was determined by mercury intrusion porosimetry (MIP, MicroActive AutoPore V, Micromeritics, USA). In addition, three-dimensional structural models of ZPS samples were analyzed using a 3D X-ray microscope (Xradia 620 Versa, Zeiss, Germany) to evaluate internal architecture and structural integrity.

Compression tests were performed using a universal testing machine (Instron 5969, USA) in accordance with ASTM E8 and ASTM E9-2009 standards. Prior to compression testing, all specimens were ultrasonically cleaned in absolute ethanol and dried at room temperature (≈20 °C). Porous samples with dimensions of 6 × 6 × 6 mm were tested at a constant crosshead speed of 2 mm min^−1^. Each test was repeated four times to obtain mean values and standard deviations.

### 2.3 In vitro degradation and ion release measurement

*In vitro* degradation tests were performed according to ASTM G31-72. Both dense and porous specimens were immersed in Hank's balanced salt solution (HBSS, pH 7.4) at 37 °C for 28 days. For porous specimens, cylindrical samples with dimensions of Φ10 × 2 mm were used. The solution volume was maintained at 20 mL per cm^2^ of exposed sample surface area. Ion release (Zn^2+^ and Li^+^) was quantified using ICP-OES at predetermined time points (1, 7, 14, 28 days). After removing corrosion products with CrO_3_ solution, the weight loss of the samples was measured using an electronic balance with an accuracy of ±0.1 mg, and the weight loss (%) was calculated. Each type of test was conducted on four samples to obtain the average value and standard deviation.

### 2.4 Liquid and blood absorption test

Samples were fully immersed in deionized water and subjected to ultrasonic treatment to facilitate complete wetting and penetration of the liquid into the porous structure. After immersion, samples were removed and gently blotted with absorbent paper to eliminate excess surface water and minimize interference from residual droplets. The dry weight and wetted weight of each sample were recorded. The absorbed liquid volume was calculated from the weight increment and the density of deionized water. Liquid absorption capacity was expressed as the ratio of absorbed liquid volume to the total sample volume. Each group was tested in quadruplicate, and results were presented as mean ± standard deviation.

Each sample was placed on an individual piece of filter paper. A 40 μL droplet of whole blood was carefully deposited onto the surface of the specimen, and timing was initiated immediately. The mass change of the filter paper was recorded at 0, 1, 3, and 5 min to evaluate blood leakage and outward diffusion from the scaffold surface. After each measurement, the position of the specimen was adjusted, and the distribution pattern of blood on the filter paper was documented to assist in the qualitative assessment of diffusion behavior.

The mass change of the samples was subsequently measured to determine blood absorption and retention capacity. Prior to weighing, loosely attached blood on the sample surface was gently removed using filter paper to reduce the influence of unbound surface droplets. All experiments were performed in quadruplicate, and results were expressed as mean ± standard deviation.

### 2.5 Isolation and culture of primary rat BMSCs

Primary BMSCs were isolated from the femurs of 4-week-old Sprague-Dawley rats. All animal procedures were approved by the local Ethics Committee for Animal Experiments (Approval No. KQYY-202507-002), and conducted in accordance with relevant guidelines. Briefly, rats were euthanized under approved protocols, and bone marrow was flushed from femurs using sterile α-MEM medium. The cell suspension was filtered through a 70 μm cell strainer and centrifuged to collect cells. The resulting cell pellet was resuspended in α-MEM supplemented with 10% fetal bovine serum and 1% penicillin/streptomycin and cultured at 37 °C in a humidified atmosphere containing 5% CO_2_. After 24 h, non-adherent cells were removed by medium replacement. Adherent cells were cultured with medium changed every two days. Cells at passages 3-5 were used for all subsequent experiments to ensure phenotypic stability.

To determine cytocompatibility under direct contact conditions, BMSCs cultured on scaffolds for 24 h were stained using a Calcein-AM/propidium iodide (PI) kit (Beyotime, China) according to manufacturer instructions. Fluorescence images were obtained using confocal microscopy. The live/dead ratio was calculated by counting green (viable) and red (non-viable) cells in five randomly selected fields per scaffold. Experiments were performed with three independent biological replicates.

### 2.6 Scaffold seeding, cell adhesion and cytoskeletal organization

BMSCs were seeded onto scaffolds at a density of 1 × 10^6^ cells per scaffold in 24-well plates. Prior to cell seeding, scaffolds were sterilized by ethylene oxide and pre-incubated in sterile α-MEM for 2 weeks at 37 °C (medium refreshed every 2-3 days) to allow complete medium infiltration and surface conditioning. After pre-incubation, scaffolds were transferred to fresh complete medium immediately before cell seeding. Cells were allowed to adhere for 4 h before adding additional medium to fully immerse the scaffolds. All *in vitro* experiments were conducted using at least three independent biological replicates.

To evaluate early adhesion behavior and mechanosensitive morphology, BMSCs cultured on scaffolds for 24 h were fixed with 4% paraformaldehyde and permeabilized with 0.1% Triton X-100. F-actin was stained using phalloidin conjugated to Alexa Fluor 594 (Beyotime, China), and nuclei were counterstained with DAPI. Confocal microscopy (Zeiss LSM series) was used to capture z-stack images to ensure accurate assessment of cell distribution along the 3D scaffold surface. Cell spreading area was quantified using ImageJ software by manually outlining cell boundaries from maximum projection images. At least 50 cells from randomly selected regions per sample were analyzed. For ultrastructural observation, additional samples were dehydrated through graded ethanol and observed via SEM to visualize filopodia extension and focal contact formation.

### 2.7 Extract preparation and proliferation assay

The extracts used for all *in vitro* assays were prepared according to ISO 10993-5 by incubating the scaffolds for 24 h; therefore, ICP measurements were performed on these 24 h extracts. Samples were immersed in complete culture medium at a ratio of 3 mL cm^−2^ and incubated at 37 °C for 24 ± 0.5 h. The extracts were filtered through a 0.22 μm membrane and diluted to 5%, 10%, and 15% concentrations for subsequent assays. BMSCs were seeded in 96-well plates at 5 × 10^3^ cells per well. After attachment, culture medium was replaced with diluted extracts. Cell proliferation was assessed at 24, 48, and 72 h using a CCK-8 assay (Beyotime, China). Absorbance at 450 nm was recorded using a microplate reader. Each condition was tested in five technical replicates and three independent experiments.

### 2.8 Osteogenic differentiation and functional assays

To induce osteogenic differentiation, BMSCs were cultured in osteogenic medium consisting of α-MEM supplemented with 10 mM β-glycerophosphate (Sigma-Aldrich, USA), 50 μg mL^−1^ ascorbic acid (Sigma-Aldrich, USA), and 100 nM dexamethasone (Sigma-Aldrich, USA). For alkaline phosphatase (ALP) activity, cells were harvested on day 5. Total protein was extracted using RIPA buffer and quantified via BCA assay. ALP activity was measured using a colorimetric kit (Nanjing Jiancheng Bioengineering Institute, China) and normalized to total protein content. For mineralization assessment, cells were stained with 2% Alizarin Red S (ARS; Sigma-Aldrich, USA) on day 14. After washing, the bound dye was dissolved in 10% cetylpyridinium chloride, and absorbance was measured at 570 nm.

### 2.9 Immunofluorescence staining and Western blotting

To determine whether groove-ridge-like textures activated focal adhesion-mediated mechanotransduction, immunofluorescence staining for VCL (vinculin) and YAP was performed after 48 h of culture. For YAP localization analysis, fluorescence intensity in nuclear and cytoplasmic regions was quantified using ImageJ. The nuclear-to-cytoplasmic (N/C) ratio was calculated from at least 80 cells per group. In this study, increased YAP N/C ratio was interpreted as enhanced YAP nuclear signaling (YAP activation). Western blotting was conducted to detect total YAP, phosphorylated YAP (Ser127), total FAK, phosphorylated FAK (Tyr397), and VCL. Protein lysates were collected after 3 days of culture to capture early mechanotransduction events. For Western blot readouts, YAP activation was evaluated using the p-YAP (Ser127)/total YAP ratio, where a lower p-YAP/YAP ratio was interpreted as increased YAP activation because Ser127 phosphorylation is associated with cytoplasmic retention. All assays were performed in triplicate with three independent biological replicates.

Western blotting was performed on BMSCs cultured on different scaffolds to examine mechanotransduction activation (day 3) and osteogenesis-related protein expression (day 7) *in vitro*. Briefly, cells were washed twice with ice-cold PBS and lysed on ice using RIPA buffer supplemented with protease and phosphatase inhibitor cocktails. Lysates were collected and centrifuged at 12,000 × g for 15 min at 4 °C to remove insoluble debris. Total protein concentration was determined using a BCA assay (Beyotime, China). Proteins were separated by SDS-PAGE and transferred onto PVDF membranes. Membranes were blocked with 5% BSA in TBST and incubated overnight at 4 °C with the indicated primary antibodies. Detailed antibody information is provided in Table S2 (Supporting Information). After washing, membranes were incubated with HRP-conjugated secondary antibodies and visualized using enhanced chemiluminescence reagents. Secondary antibodies are listed in Table S2 as well. Band intensities were quantified using ImageJ. Protein expression levels were normalized to GAPDH, and phosphorylation levels were additionally normalized to their corresponding total protein levels. All experiments were performed with at least three independent biological replicates.

### 2.10 RNA extraction and RT-qPCR

To evaluate gene expression associated with osteogenesis and epigenetic regulation, total RNA was extracted from BMSCs using TRIzol reagent (Invitrogen) according to the manufacturer's protocol. RNA purity and concentration were assessed by spectrophotometry, and 1 μg of total RNA was reverse-transcribed into cDNA. Quantitative PCR was performed using SYBR Green master mix (CWBio, China) on a real-time PCR system (Bio-Rad, Hercules, USA). Each reaction was conducted in triplicate technical replicates, and no-template controls were included. Melting curve analysis was performed to confirm amplification specificity. Relative gene expression was calculated using the 2^−ΔΔCt^ method and normalized to β-actin. Primer sequences used in this study are listed in Table S1 (Supporting Information). All experiments were repeated with at least three independent biological replicates.

### 2.11 In vivo test and characterization

All animal procedures were approved by the local Ethics Committee for Animal Experiments (Approval No. KQYY-202507-002) and conducted in accordance with relevant guidelines. Thirty-six healthy male New Zealand White rabbits (2.5-3.0 kg) were used. Rabbits were assigned to three cohorts (Blank, Zn-0.4Li, and Ti) and two endpoints (4 and 8 weeks), with six rabbits per cohort at each time point. Rabbits were anesthetized with intravenous 3% pentobarbital sodium (30 mg kg^−1^). After shaving and disinfecting the calvarial region, a midline incision was made to expose the parietal bone. Three full-thickness circular defects (9 mm diameter) were created using a trephine bur under continuous sterile saline irrigation to avoid thermal necrosis. The defect size was defined as critical-sized according to established rabbit calvarial models. In the Blank cohort, all three defects were left empty. In the Zn-0.4Li cohort, each rabbit received three Zn-0.4Li scaffolds with different Hs values (70, 220, and 250 μm; one scaffold per defect). In the Ti cohort, each rabbit received three Ti scaffolds with the corresponding 70, 220, and 250 μm designs. Scaffold allocation to defect position was randomized within each rabbit. Scaffolds were press-fitted into the defect without additional fixation. The periosteum and skin were sutured in layers. Postoperative antibiotic prophylaxis (penicillin 40,000 IU kg^−1^) was administered for three consecutive days. Animals were monitored daily. Animals were euthanized at 4 or 8 weeks. After euthanasia, the calvarial specimens were harvested en bloc and first subjected to micro-CT analysis. The same harvested specimens were then processed for subsequent histological and immunofluorescence analyses using the corresponding tissue sections.

At 4 and 8 weeks, calvarial specimens were harvested and fixed in 4% paraformaldehyde for 48 h. Samples were scanned by SkyScan 1276 (Bruker SkyScan, Aartselaar, Belgium). The parameters were set as follows: Source Voltage (kV) = 80, Source Current (μA) = 200, image pixel size (μm) = 7.042090, depth (bits) = 16, reference intensity = 58,000, exposure (ms) = 600 and rotation = Step (deg) = 0.400. Images were reconstructed using a three-dimensionally reconstructed software program (CTVox, Bruker). Quantification was performed using CTAn software (Bruker, Belgium). All analyses were conducted in a blinded manner. Specimens were embedded in methyl methacrylate under vacuum infiltration and polymerized at low temperature. Sections (40-60 μm) were prepared using a precision diamond saw. Sections were stained with 1% methylene blue followed by 0.1% acid fuchsin. Alizarin Red S (Sigma-Aldrich, USA) and Calcein (Sigma-Aldrich, USA) were administered at weeks 1 and 3, respectively. Mineral apposition rate (MAR) was calculated by measuring the distance between fluorescent labels divided by time interval.

Immunofluorescence staining was performed on decalcified calvarial sections to evaluate mechanotransduction and osteogenic/epigenetic markers in regenerated tissue. After harvesting, specimens were fixed in 4% paraformaldehyde at 4 °C for 48 h and subsequently decalcified in 10% (w/v) EDTA solution (pH 7.2-7.4) at room temperature for 6 weeks with gentle agitation; the EDTA solution was refreshed every 2-3 days to maintain decalcification efficiency. Following decalcification, samples were rinsed thoroughly in running water, dehydrated through a graded ethanol series, cleared in xylene, and embedded in paraffin. Serial sections (5 μm) were obtained using a rotary microtome. Sections were deparaffinized, rehydrated, and subjected to antigen retrieval (citrate buffer, pH 6.0) under controlled heating conditions. After cooling to room temperature, sections were permeabilized (0.1% Triton X-100, if required) and blocked with 5% bovine serum albumin for 1 h to minimize nonspecific binding. Sections were incubated with primary antibodies against p-YAP (Ser127), total YAP, RUNX2, OSX, KDM5A, and KDM6A overnight at 4 °C, followed by incubation with fluorophore-conjugated secondary antibodies for 1 h at room temperature in the dark. Detailed antibodies used for immunofluorescence staining are listed in Table S3 (Supporting Information). Nuclei were counterstained with DAPI. Fluorescence intensity was quantified using ImageJ. Mean fluorescence intensity was normalized to DAPI-positive nuclei. For YAP activation assessment, the p-YAP/YAP ratio was calculated, with a lower p-YAP/YAP ratio interpreted as increased YAP activation (dephosphorylation-associated nuclear signaling). Five random regions within the region of interest (ROI) were analyzed per sample.

### 2.12 Statistical analysis

All data are presented as mean ± standard deviation. Normality was assessed using Shapiro-Wilk test. When data met normality assumptions, parametric tests were used, including t tests for two-group comparisons and one-way or two-way ANOVA with Tukey's post hoc test for multiple comparisons, as appropriate. When data did not meet normality assumptions, nonparametric tests were used, including Mann-Whitney U tests for two-group comparisons and Kruskal-Wallis tests with post hoc multiple comparisons for multiple independent groups. A two-sided *p* < 0.05 was considered statistically significant. Significance is denoted as follows: not significant (ns) *p* > 0.05, *p < 0.05, **p < 0.01, ***p < 0.001.

## 3 Results

### 3.1 Laser scanning strategy modulates surface texture and physical performance

**Fig. 2:**
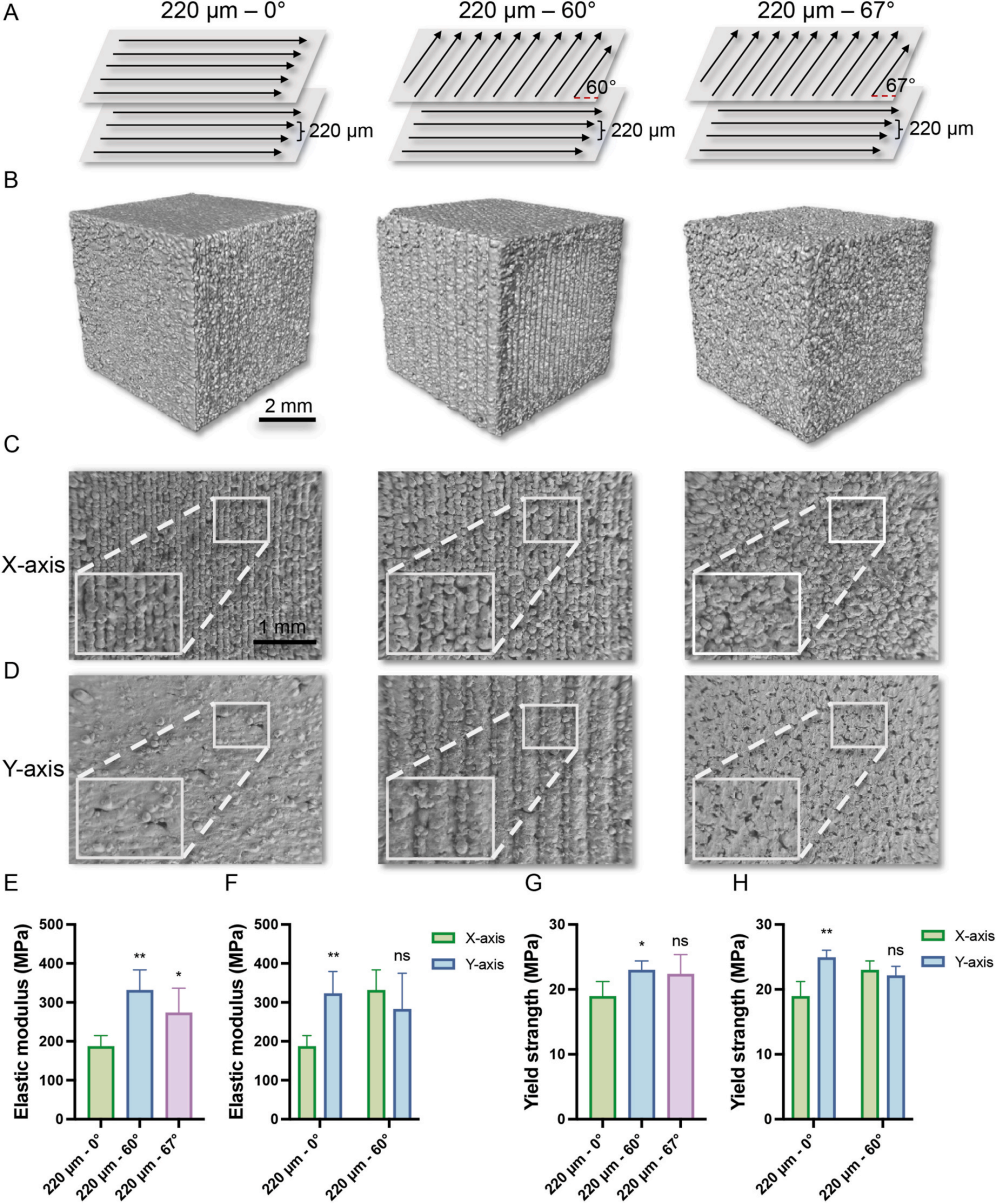
Programming groove-ridge-like textures by interlayer rotation. (A) Scanning strategy; (B) Micro-CT characterization of specimens fabricated with different rotation angles; (C, D) SEM images of the X- and Y-direction surfaces for specimens fabricated with different rotation angles; (E) Elastic modulus of specimens fabricated with different rotation angles; (F) Direction-dependent Elastic modulus of the 0° and 60° groups (X vs. Y); (G) Yield strength of specimens fabricated with different rotation angles; (H) Direction-dependent Yield strength of the 0° and 60° groups (X vs. Y). Data are presented as mean ± SD. In panels E and G, statistical significance was determined relative to the 220 μm-0° group; in panels F and H, statistical significance was determined relative to the corresponding X-axis group.

In L-PBF, customized laser energy input induces micropores, whose spatially regular stacking can be modulated by the scanning strategy to form distinct microarchitecture on the surface and interior. Fig. 2 shows the influence of interlayer rotation angles on the surface morphology and mechanical properties. The 0° group exhibited groove-ridge-like features along X and rough surfaces along Y, indicating pronounced anisotropy. The 60° group showed uniformly distributed groove-ridge-like textures in both directions, whereas the 67° group displayed rough, porous surfaces without distinct patterns (Fig. 2A–D). These differences can be attributed to the stacking behavior of the micropores: under 0° rotation, they accumulate along a single direction; under 60° rotation, they interlace between adjacent layers; and under 67° rotation, they become more dispersed, thereby preventing the formation of continuous groove-ridge-like textures.

Mechanical testing (Fig. 2E–H) mirrored these trends. Increasing the rotation from 0° to 60° markedly enhanced elastic modulus and yield strength, while further increasing to 67° caused a slight decline, yet values remained above those of the 0° group. At 0°, aligned micropores created directional stress concentrations, reducing stiffness and producing anisotropy. At 60°, interlaced defects formed a more uniform load-bearing network, improving both modulus and strength and minimizing directional differences. At 67°, defect dispersion slightly lowered stiffness, but overall performance remained relatively high. In summary, the 60° interlayer rotation optimizes micropore stacking, enhances uniformity of load-bearing pathways, improves mechanical performance, and reduces structural anisotropy. All subsequent experiments employed scaffolds with this rotation angle, and the Hs was varied in subsequent experiments.

**Fig. 3:**
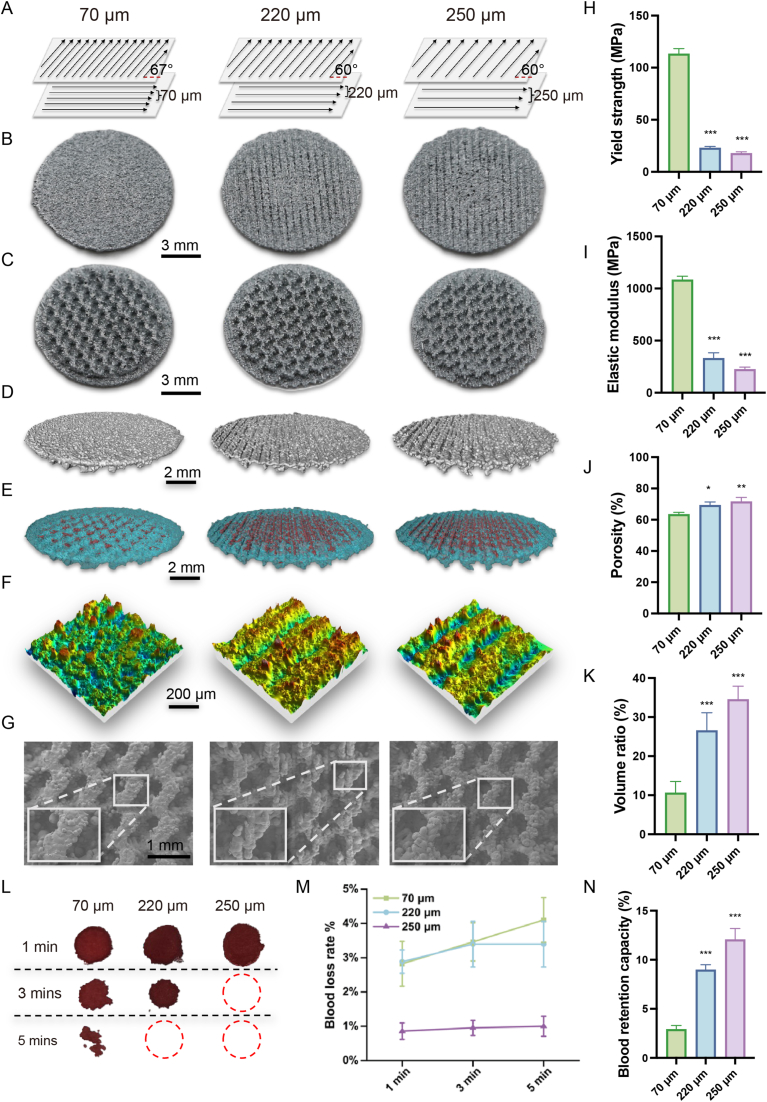
*In situ* formation of groove-ridge-like textures on TPMS pore walls by Hs. (A) Scanning strategy; (B) Macroscopic front view of the as-printed specimens; (C) Macroscopic back view of the as-printed specimens; (D) Micro-CT reconstruction/characterization; (E) Porosity of inherent micropores; (F) 3D surface topography profiling; (G) SEM characterization of surface morphology; (H) Yield strength; (I) Elastic modulus; (J) Total porosity; (K) Liquid absorption analysis; (L) Representative images showing blood leakage at 1, 3, and 5 min for different scaffolds (from top to bottom); (M) Quantification of blood loss rate derived from filter-paper mass increase; (N) Blood retention capacity of scaffolds after gently removing non-adherent blood. The 220 μm and 250 μm groups are defined as the Osteogenic Conditioning Scaffold (OCS), and the 70 μm group serves as the TPMS control. Data are presented as mean ± SD. Statistical significance was determined relative to the 70 μm group.

Regarding Hs, the emergence of groove-ridge-like textures may contribute to improved scaffold biocompatibility. Live/dead staining further confirmed that the 220 and 250 μm groups exhibited the best cellular responses (Fig. S1A and S1B), although the 70 and 100 μm groups showed higher Young's modulus and yield strength (Fig. S1C and S1D). During preliminary screening, the 100 μm group showed limited microtexture differences from the 70 μm group, whereas the 280 μm group exhibited poor structural integrity after fabrication. Therefore, considering the trade-off between mechanical properties, cytocompatibility, and structural integrity, three representative scaffolds (Hs = 70, 220, and 250 μm) were selected for subsequent analysis. Scaffold selection prioritized an overall balance among mechanical support, transport-related properties, and biological performance, rather than maximizing stiffness alone. As Hs increased from 70 to 220 and 250 μm, groove-ridge-like textures gradually emerged, as revealed by SEM and 3D micro-CT reconstructions (Fig. 3A–D). Surface profilometry confirmed the trend, showing smooth, dense surfaces at 70 μm and distinct periodic undulations at 220 and 250 μm. Confocal surface profiling further supported this observation (Fig. S7). The 70 μm group showed relatively small, non-directional surface undulations, whereas the 220 μm and 250 μm groups displayed continuous groove-ridge-like features with more pronounced height variations. Representative line profiles showed that the characteristic width of these features increased with Hs, and quantitative roughness analysis revealed an increasing trend in Sa. These results further indicate that larger Hs reduced melt-track overlap and promoted the formation of a more directional pore-wall microtexture. Porosity analysis (Fig. 3E and J) indicated that larger Hs increased both interior porosity and pore interconnectivity, generating numerous fine, interconnected micropores. At 250 μm, however, structural inhomogeneity and partial collapse led to blurred features and reduced mechanical stability, indicating that excessive Hs may compromise scaffold integrity. Mechanical testing (Fig. 3H and I) showed that the 70 μm group had the highest elastic modulus and yield strength due to higher densification, whereas the 220 μm and 250 μm groups exhibited lower stiffness but retained overall mechanical integrity.

In contrast, the increased interior porosity enhanced fluid absorption. Liquid absorption capacity rose from ∼10.6% at 70 μm to ∼26.6% at 220 μm and ∼34.5% at 250 μm (Fig. 3K), accompanied by improved blood absorption and retention (Fig. 3L–N). Blood leakage and spreading decreased, blood loss rate declined, and retention increased from ∼2.9% at 70 μm to ∼12% at 250 μm. These results indicate that the OCS groups (220 and 250 μm) exhibited improved wettability and fluid retention within the micropore network, offering a more stable microenvironment for early cell colonization. Consistently, protein adsorption assays showed higher serum protein adsorption on the OCS groups (220 and 250 μm) than on the 70 μm group at both 4 h and 24 h (Fig. S6), providing additional evidence for improved interfacial protein retention.

### 3.2 Scaffold extracts differentially regulate BMSC osteogenic responses

**Fig. 4:**
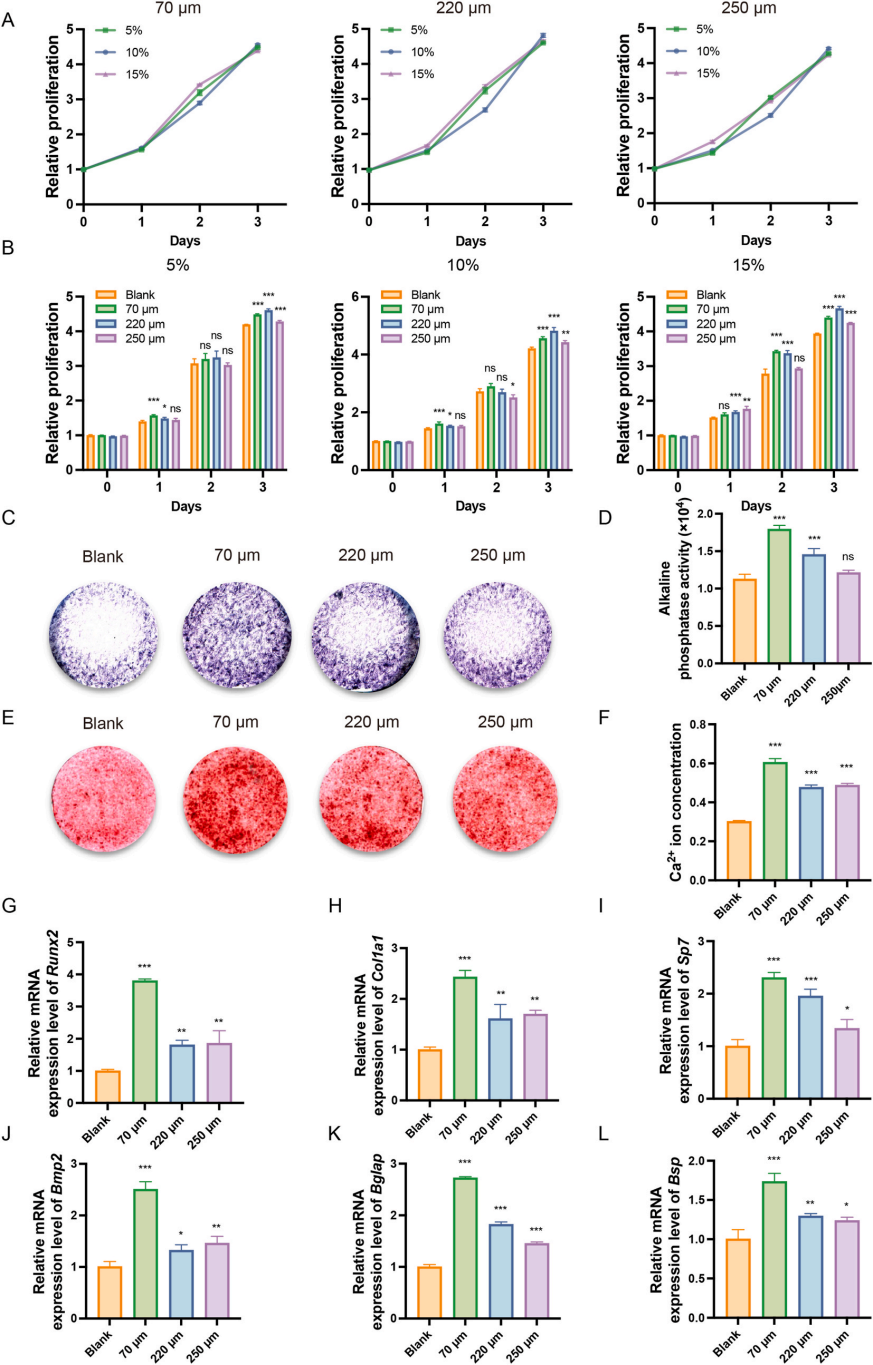
Cytocompatibility and pro-osteogenic effects of scaffold extracts from the OCS (220 and 250 μm groups) and the TPMS control (70 μm group). (A, B) Proliferation curves of BMSCs cultured with scaffold extracts at the indicated dilution ratios; (C, D) ALP staining and quantitative analysis; (E, F) Alizarin Red S staining and quantitative analysis; (G-L) RT-qPCR analysis of osteogenesis-related gene expression and corresponding quantification. Data are presented as mean ± SD. Statistical significance was determined relative to the blank group.

Extracts from all groups exhibited no obvious cytotoxicity at 5-15% dilutions (Fig. 4A and B) and promoted cell proliferation compared with the blank group. Notably, at the same dilution ratio, extracts derived from the 70 μm and 220 μm scaffolds stimulated BMSC proliferation more markedly, whereas the proliferation rate in the 250 μm extract group was comparable to that of the blank group. Further osteogenic assays reinforced this trend. Compared with the blank group, all extract-treated groups showed enhanced ALP activity and mineral deposition (Fig. 4C–F), and upregulated osteogenesis-related genes (Fig. 4G–L) with the 70 μm extract group exhibiting the most pronounced effects. Notably, the extract assay was used here to probe the contribution of released species under defined dilution conditions, rather than to rank scaffold performance under direct contact or *in vivo* settings. These results suggest that released species from the 70 μm scaffold can contribute to stronger osteogenic readouts in extract assays under the tested dilution conditions.

This difference may be associated with differences in released-species profiles among the extracts. Ion concentration measurements showed that Zn^2+^ concentrations tended to increase with Hs, although no significant differences were detected among the 70, 220, and 250 μm groups under the tested extract conditions (Fig. S8A). In contrast, Li^+^ concentrations were significantly higher in the 220 and 250 μm groups than in the 70 μm group (Fig. S8B). These data indicate that the scaffold extracts differed in their released-species profiles, particularly in Li^+^ release, which may contribute to the differential osteogenic responses observed in the extract assays.

### 3.3 OCS enhances BMSC adhesion, spreading, and osteogenic differentiation

**Fig. 5:**
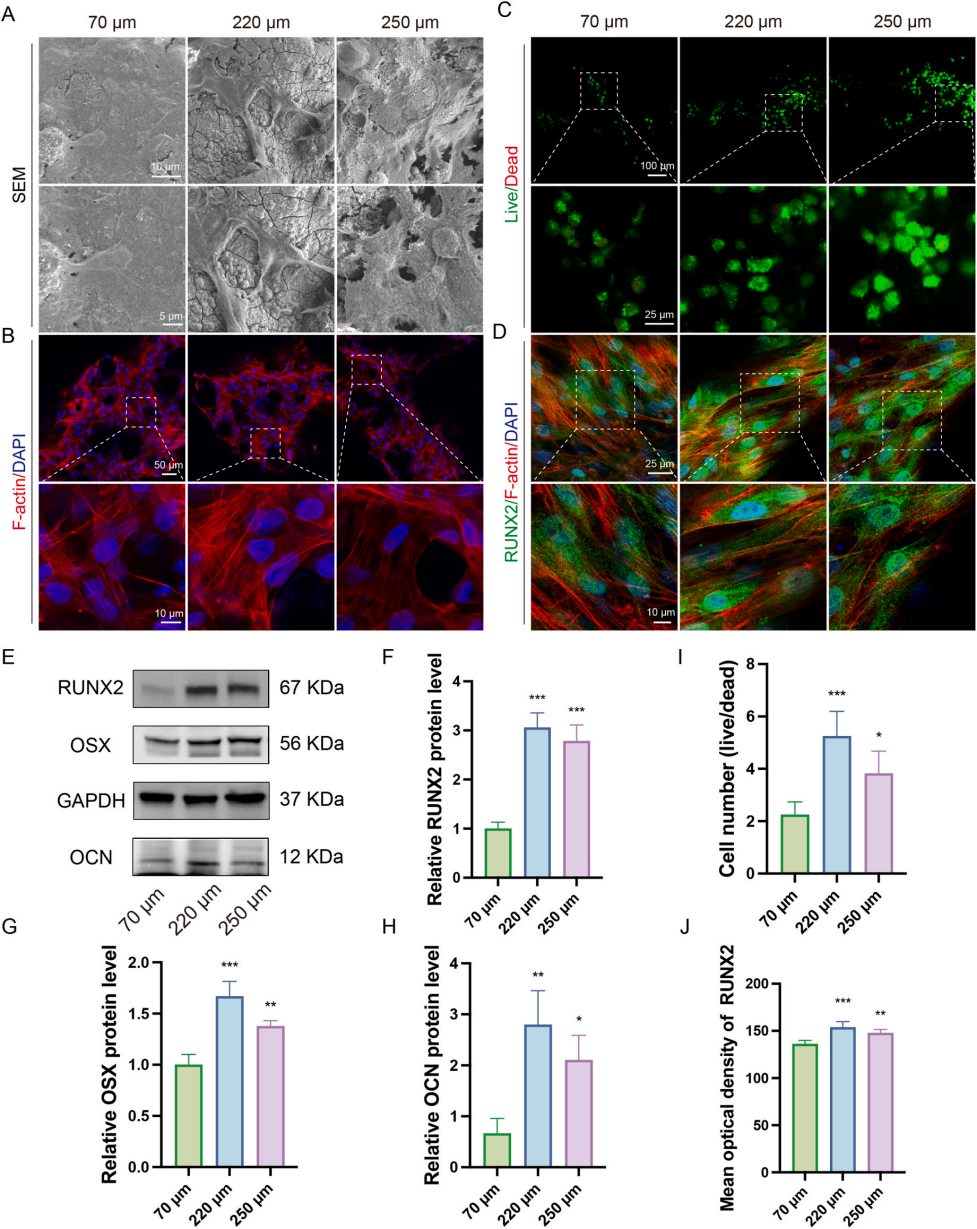
Effects of different scaffolds on BMSC adhesion and osteogenic differentiation. (A) Representative SEM images of BMSCs on the scaffolds after 24 h; (B) cytoskeletal staining of BMSCs after 24 h (F-actin, red; nuclei, DAPI, blue); (C) live/dead staining of BMSCs after 24 h (live cells, green; dead cells, red); (D) immunofluorescence staining of BMSCs on the scaffolds (RUNX2, green; F-actin, red; nuclei, DAPI, blue); (E-H) Western blotting of osteogenic-related proteins and corresponding quantification; (I) quantitative analysis of live/dead staining; (J) quantitative analysis of immunofluorescence staining. Data are presented as mean ± SD. Statistical significance was determined relative to the 70 μm group.

The 70 μm scaffolds exhibited relatively smooth surfaces with fewer surface deposits observed by SEM, where BMSCs appeared contracted with short filopodia. In contrast, the OCS featured rougher surfaces, supporting larger, well-spread cells with abundant branched filopodia firmly anchored to the scaffold (Fig. 5A). F-actin staining confirmed a more extensive and organized cytoskeleton on the OCS groups (Fig. 5B), consistent with enhanced adhesion and viability. Live/dead assays aligned with these observations: the 70 μm group showed the lowest live/dead ratio, while the OCS supported higher cell survival, with the 220 μm group showing the most favorable results (Fig. 5C and I).

Building on early cell attachment, the scaffolds were further evaluated for their osteoinductive effects on the BMSCs by examining osteogenic markers via RUNX2/F-actin immunofluorescence staining and Western blotting. As an early osteogenic marker, RUNX2 expression was increased in the OCS groups, with the highest level observed in the 220 μm group (Fig. 5D and J), indicating a stronger early osteogenic response under direct-contact culture.

Late-stage osteogenic markers, including Osterix (OSX) and osteocalcin (OCN), were strongly upregulated on OCS, with the 220 μm group showing the highest expression of both markers (Fig. 5E–H). Together, these results indicate that OCS created a microenvironment that not only supported BMSC adhesion, spreading, and mechanotransduction but also robustly enhanced osteogenic differentiation. Notably, the 220 μm scaffold consistently showed the strongest expression of both early- and late-stage osteogenic markers. This outcome is interpreted as reflecting a favorable balance among pore-wall microtexture, microporosity/interconnectivity, fluid transport-related properties, mechanical support, degradation behavior, and released-species profiles, rather than the effect of any single scaffold characteristic.

### 3.4 OCS enhances VCL-FAK-YAP mechanotransduction and is accompanied by changes in KDM5A/KDM6A expression

Robust adhesion and mechanotransduction are critical not only for cell survival and proliferation but also for steering stem cell fate [[22], [23], [24], [25]]. The scaffold's microstructural features can transmit biophysical cues that reorganize the cytoskeleton, modulate epigenetic states, and ultimately drive osteogenic differentiation [26]. Given that the VCL-FAK-YAP signaling axis serves as a central pathway linking cell adhesion and cytoskeletal tension to nuclear mechanotransduction, we examined its activation in cells cultured on the scaffold surface.

**Fig. 6:**
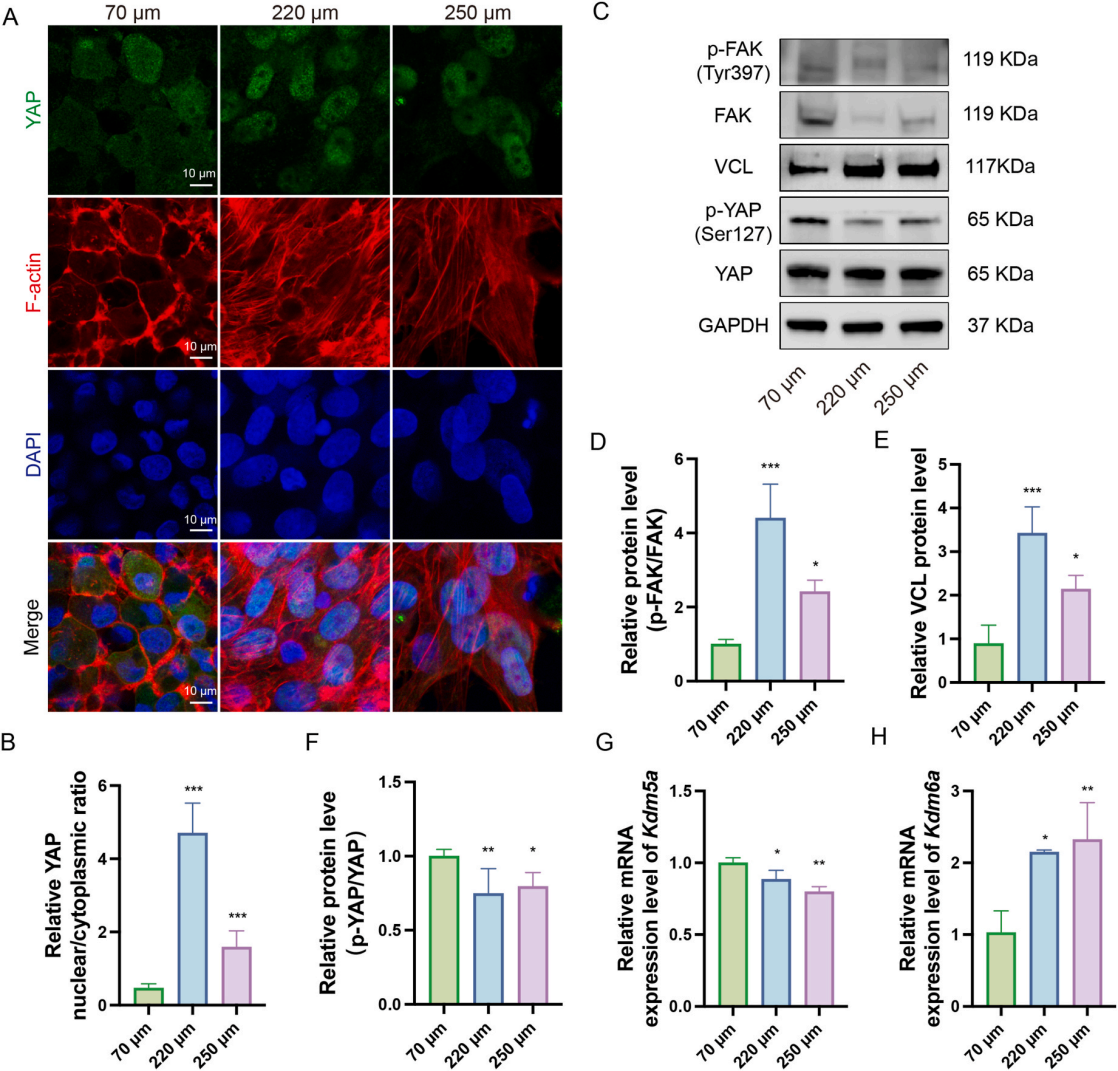
Effects of different scaffolds on mechanotransduction-associated signaling and KDM5A/KDM6A expression in BMSCs. (A, B) Immunofluorescence staining and quantitative analysis (YAP, green; F-actin, red; nuclei, DAPI, blue); (C-F) Western blotting and corresponding quantification; (G, H) RT-qPCR analysis and corresponding quantification. Data are presented as mean ± SD. Statistical significance was determined relative to the 70 μm group.

VCL expression was significantly upregulated, accompanied by elevated FAK phosphorylation (Fig. 6C–F), indicating enhanced focal adhesion-associated signaling in BMSCs cultured on the OCS surfaces. In addition, increased YAP nuclear localization was observed in the OCS groups (Fig. 6A and B), indicating enhanced mechanotransduction-associated signaling under direct-contact culture. Likewise, the coordinated increase in VCL expression and YAP nuclear localization explains the more spread morphology, organized cytoskeleton, and higher live/dead ratios on OCS groups. In contrast, cells on the 70 μm scaffold showed less spreading, lower viability, and weaker mechanotransduction-associated signals. Overall, these results show that the direct-contact microenvironment of the OCS groups was associated with enhanced cell spreading, focal adhesion organization, and mechanotransduction.

KDM5A, an H3K4 demethylase, when downregulated, reduces H3K4 demethylation, maintaining a transcriptionally permissive chromatin state at osteogenic gene promoters such as RUNX2 and OSX [27,28]. Conversely, KDM6A upregulation decreases H3K27me3, relieving repression of osteogenic genes [29,30]. RT-qPCR revealed downregulation of *Kdm5a* and upregulation of *Kdm6a* in the OCS groups (Fig. 6G and H).

### 3.5 OCS accelerates bone regeneration and osseointegration in a rabbit calvarial defect model

**Fig. 7:**
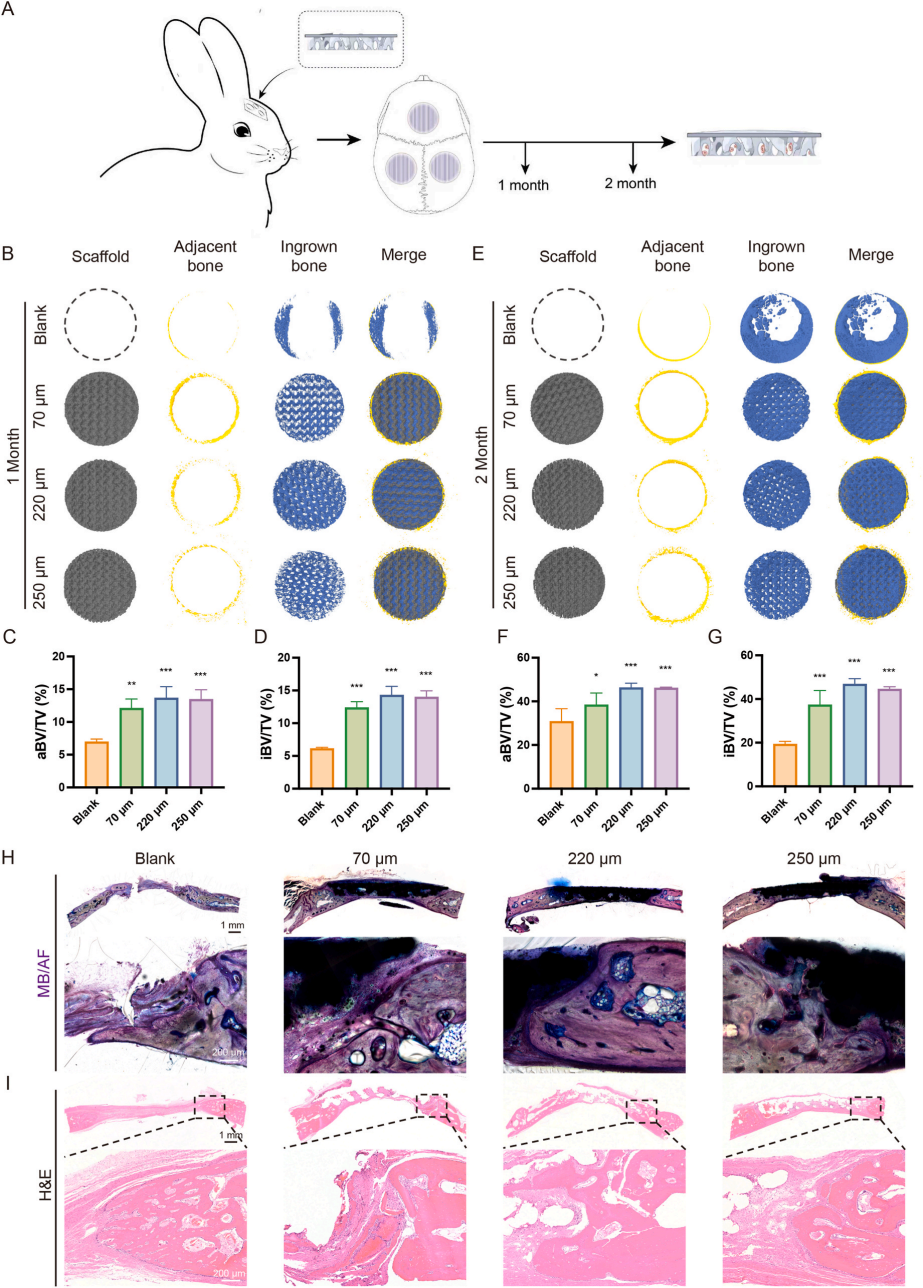
Bone regeneration and osseointegration in a rabbit calvarial defect model. (A) Schematic illustration of the calvarial defect model; representative 3D micro-CT reconstructions (B), BV/TV of newly formed bone around the scaffolds (C) and within the defect region (D) at 1 month post-implantation; representative 3D micro-CT reconstructions (E), BV/TV of newly formed bone around the scaffolds (F), and within the defect region (G) at 2 months post-implantation; (H) methylene blue/acid fuchsin (MB/AF) staining at 1 month post-implantation; (I) H&E staining at 1 month post-implantation. Data are presented as mean ± SD. Statistical significance was determined relative to the blank group.

Although the OCS groups demonstrated enhanced mechanotransductive responses in BMSCs, their *in vivo* osteogenic potential required further validation. Thus, a rabbit critical-sized calvarial defect model was established (Fig. 7A).

At 1 month, 3D micro-CT reconstruction showed minimal bone formation at the defect margins in the blank group. In contrast, all Zn-0.4Li scaffold groups exhibited markedly enhanced new bone formation within the defect (Fig. 7B). Notably, the OCS groups further accelerated bone regeneration, with the 220 μm group showing the most prominent effect. Quantitative analysis revealed significantly higher BV/TV in the adjacent bone for the OCS groups compared with the blank and 70 μm groups, with the 220 μm group slightly outperforming the 250 μm group (Fig. 7C). A similar trend was observed within the defect core, where the 220 μm group demonstrated the most robust bone ingrowth, highlighting its superior regenerative potential (Fig. 7D).

At 2 months, 3D micro-CT reconstructions indicated increased bone formation in all groups compared with the 1-month time point. In the 220 μm group, newly formed bone almost filled the defect area, and the boundary between peripheral bone and the scaffold became less distinguishable, suggesting improved osseointegration. The 250 μm group exhibited a modest increase in new bone volume, while the scaffold appeared slightly loosened due to degradation. In the 70 μm group, new bone remained mainly confined to the defect margins, with limited bone formation in the central region (Fig. 7E). Quantitatively, BV/TV values increased in all groups at 2 months relative to 1 month; the 220 μm group maintained the highest BV/TV both around the scaffold and within the defect, and the difference compared with the 250 μm group became more evident. Although the blank group showed an increase, bone volume remained significantly lower than that of the 220 μm group (Fig. 7F and G). Collectively, these *in vivo* findings were highly consistent with our *in vitro* observations.

Undecalcified sections stained with methylene blue/acid fuchsin (MB/AF) showed evident bone healing in all scaffold-implanted groups. More pronounced scaffold degradation was observed in OCS groups, and the newly formed lamellar bone within the defect appeared thicker than that in the 70 μm group. In the blank group, only a small amount of newly formed tissue was observed at the defect edges. In the 70 μm group, the defect area was dominated by fibrous connective tissue and osteoid-like tissue with only sparse bone-like structures. In the 220 μm group, abundant newly formed bone tissue was observed and exhibited intimate integration with the scaffold interface. Although bone formation was also detected in the 250 μm group, the newly formed bone was more dispersed and the scaffold integrity was slightly compromised (Fig. 7H). H&E staining further demonstrated that the 220 μm group presented the most mature and abundant trabecular bone in the defect region, whereas the blank group showed negligible bone formation; trabeculae in the 70 μm and 250 μm groups were relatively sparse with a higher proportion of fibrous tissue (Fig. 7I). In addition, H&E staining of major organs at 8 weeks postoperatively showed preserved overall tissue architecture in the lung, heart, liver, spleen, and kidney of the Zn-0.4Li scaffold group, with no obvious tissue necrosis, hemorrhage, severe inflammatory infiltration, or structural disruption under the tested implantation conditions (Fig. S9).

**Fig. 8:**
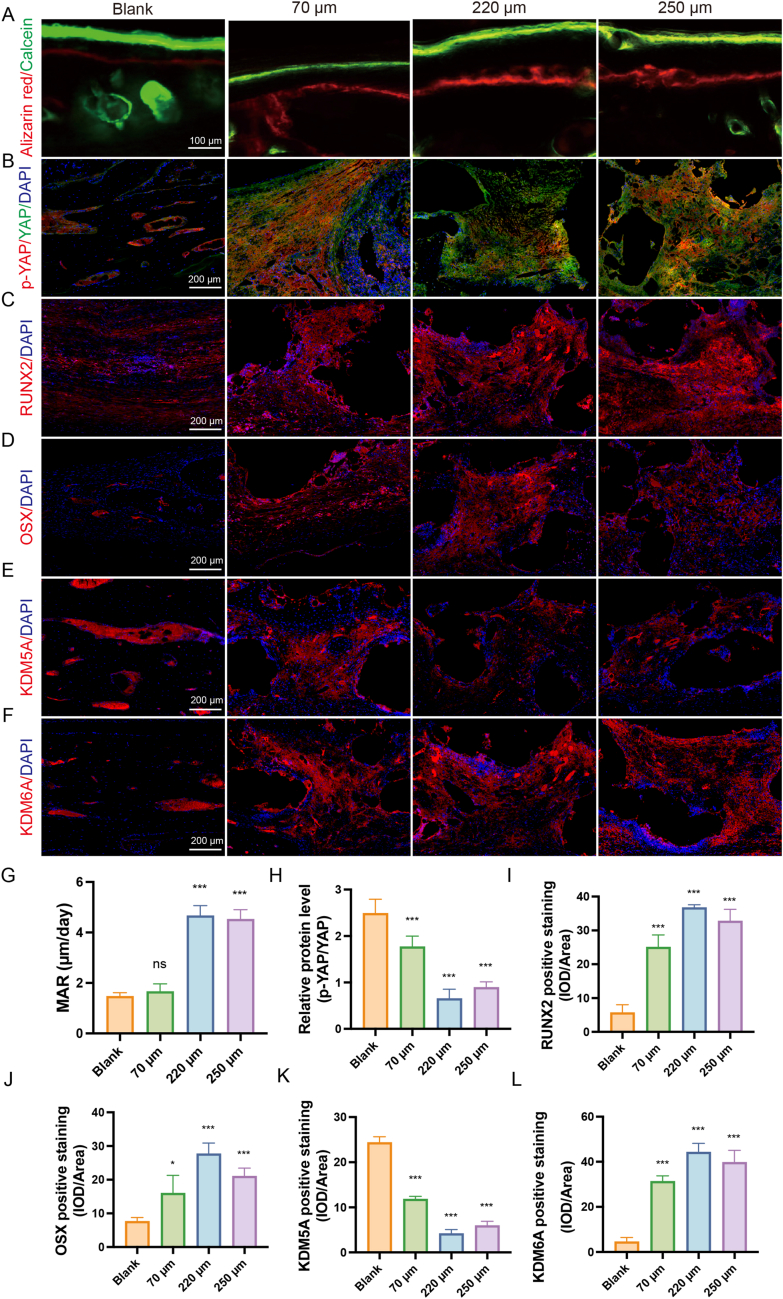
Histology of calvarial defect repair at 1 month. (A) Sequential fluorochrome labeling with alizarin red and calcein; (B-F) immunofluorescence staining of regenerated tissue within the defect region; (G) quantification of mineral apposition rate (MAR); (H-L) semiquantitative analysis of immunofluorescence staining. Data are presented as mean ± SD. Statistical significance was determined relative to the blank group.

Dynamic histomorphometry using sequential fluorochrome labeling (alizarin red, red; calcein, green) was performed to assess the MAR. The distance between the red and green labels represents the amount of newly deposited bone over the labeling interval. The 220 μm group exhibited the highest MAR, indicating accelerated local bone deposition in the optimized OCS group (Fig. 8A and G). Immunofluorescence staining was further conducted to examine mechanotransduction and osteogenic markers in regenerated tissue. The 220 μm group exhibited the lowest p-YAP/YAP ratio, suggesting enhanced YAP activation and mechanotransduction-associated responses in defect-associated cells within the optimized OCS group (Fig. 8B and H). In parallel, osteogenic markers RUNX2 and OSX were upregulated in the 220 μm group, indicating more robust activation of osteogenic transcriptional programs (Fig. 8C, D, 8I, 8J). Moreover, epigenetic markers were assessed *in vivo*: the 220 μm group showed downregulation of KDM5A and upregulation of KDM6A, consistent with the *in vitro* findings (Fig. 8E, F, 8K, 8L). Collectively, the OCS groups exhibited enhanced bone formation, mineralization, and mechanotransduction-associated signaling, together with changes in KDM5A and KDM6A expression. Notably, Ti scaffolds with the same 70, 220, and 250 μm structural designs showed a similar Hs-dependent *in vivo* trend (Figs. S2 and S3), supporting a contribution of scaffold structural design to bone regeneration in the absence of Zn^2+^/Li^+^ release. However, these results do not isolate the effect of pore-wall texture from other Hs-dependent structural and physical changes.

## 4 Discussion

In large bone defects, insufficient adhesion and osteogenic capacity of autologous mesenchymal stem cells often lead to inadequate bone regeneration in the defect center. To overcome these challenges, we developed a 3D-printed Zn-0.4Li OCS with groove-ridge-like textures, designed to enhance cell function and bone regeneration while promoting effective multi-stage osseointegration. By incorporating ordered groove-ridge-like textures onto the internal pore-wall surfaces, the scaffold integrates macroporous channels for nutrient exchange and tissue ingrowth with directional interfacial cues for cell anchorage and contact guidance [31,32].

L-PBF parameters have a significant impact on the densification of printed metals, and can be used to produce interior micropores [33]. However, biodegradable zinc alloys face challenges in achieving stable formation of groove-ridge-like textures by adjusting L-PBF parameters due to their low melting point, high melt-pool fluidity, and high thermal conductivity [[33], [34], [35], [36]]. Through iterative exploration and optimization, we found that by setting the Hs above 220 μm and a 60° interlayer rotation with controlled energy distribution, groove-ridge-like textures could be constructed within the inner surfaces of complex porous channels without post-processing, forming what we termed the OCS, providing a new avenue for more effective structural design. The 60° interlayer rotation also reduced the mechanical anisotropy of the OCS, as reflected by smaller direction-dependent differences in elastic modulus and yield strength. This more isotropic response may help distribute stresses more uniformly under multi-directional loading and reduce spatial heterogeneity in mechanical cues sensed by cells [[37], [38], [39], [40]].

**Fig. 9:**
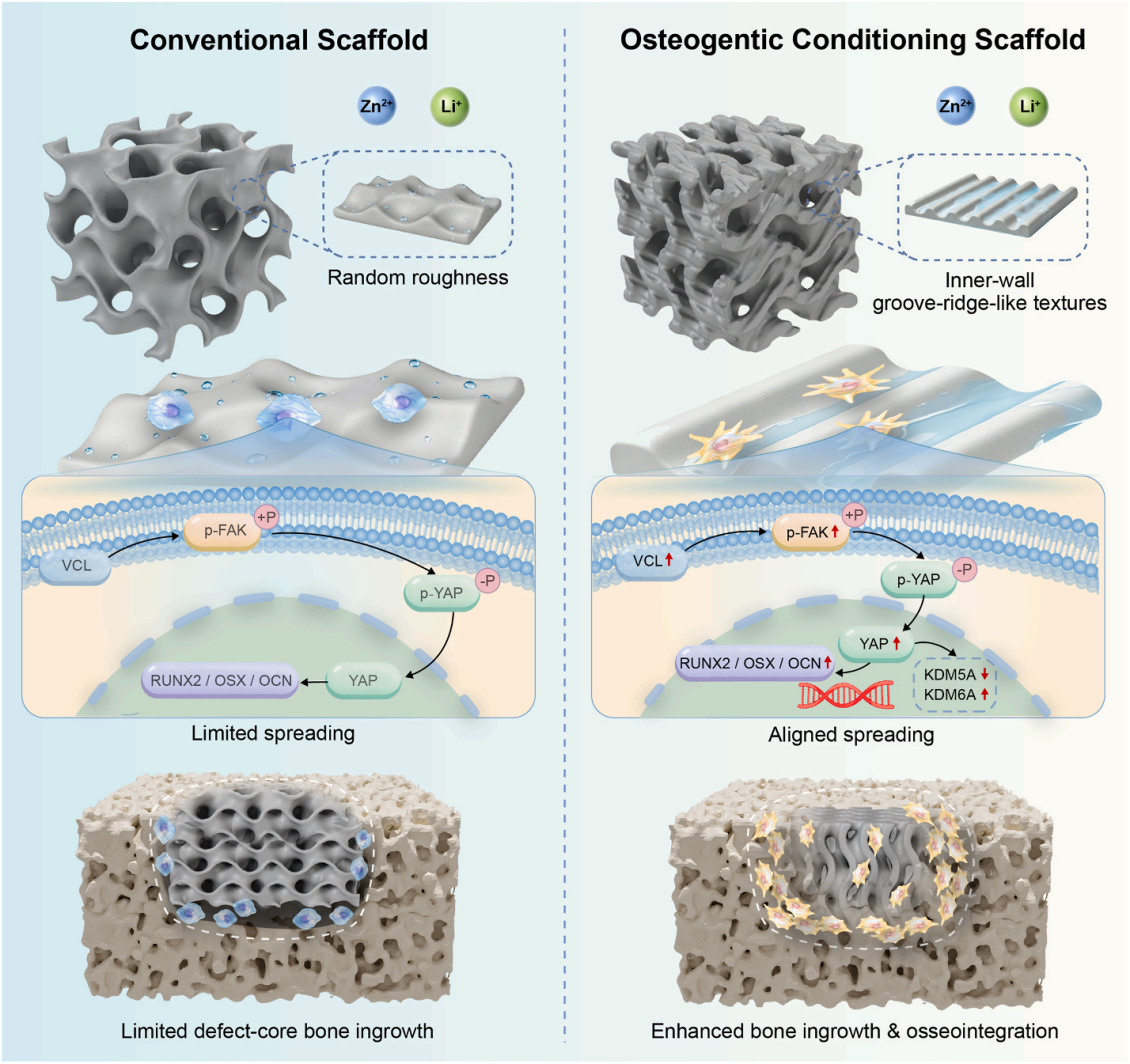
Mechanistic comparison of conventional scaffolds and the Osteogenic Conditioning Scaffold (OCS) during bone repair. This figure is a schematic representation based on findings from the present study.

The ordered groove-ridge-like textures were accompanied by improved interfacial wettability and a more favorable surface microenvironment, which together may support robust cellular responses. As presented in Fig. 9, distinct from conventional scaffolds, the unique groove-ridge-like textures of OCS efficiently facilitated the adhesion and spreading of BMSCs. These changes were accompanied by higher VCL expression, increased FAK phosphorylation, and enhanced YAP nuclear signaling, together with osteogenic marker upregulation and shifts in KDM5A and KDM6A expression. In the rabbit critical-sized calvarial defect model, OCS yielded tighter marginal osseointegration and more extensive bone ingrowth inside the defect region, resulting in markedly enhanced new bone formation compared with conventional counterparts. Importantly, immunofluorescence assays performed on degraded scaffolds *in vivo* revealed sustained YAP activation as well as KDM5A/KDM6A expression patterns consistent with *in vitro* findings. Although the pore-wall microtexture may evolve during degradation, mechanotransduction-associated signals remained detectable *in vivo* at 1 month, suggesting functional persistence of interfacial guidance cues during the early healing phase. To assess translational potential to load-bearing sites, future work will evaluate OCS in load-bearing long-bone defect models and combine time-resolved degradation analysis with post-degradation surface topography and cell-guidance assays to directly determine how guidance cues evolve during degradation.

As shown in this work, surface textures are one of several interfacial factors that regulate interfacial performance and downstream biological responses. Over the years, researchers have established a wide range of surface modification methods to improve interfacial morphologies. The reported interfacial morphologies generally include randomly distributed roughness [41], ordered regular patterns [42], and hierarchical composite architectures [43]. For biodegradable zinc alloy scaffolds, sandblasting or chemical etching is commonly used to generate random textures. Although these approaches increase specific surface area and provide attachment sites, the lack of directional cues limits stable guidance of cell behaviors, and osteogenic gains are often modest in the absence of exogenous growth factors [44]. In contrast, the ordered groove-ridge-like textures integrate biomimetic directional cues with robust engineering controllability. Although the stripe pitch (220-250 μm) exceeds the size of a single BMSC, contact guidance does not require one-to-one matching between a full cell body and one stripe period [45]. Cells sense local geometry through micron-scale focal adhesions and filopodia, which interact with ridge edges and groove slopes within a single-cell footprint. The groove-ridge interface provides continuous adhesive tracks and anisotropic mechanical gradients that spatially organize focal adhesions and aligned actin stress fibers, thereby promoting VCL-FAK activation and YAP nuclear translocation [46]. Consequently, even when the feature spacing is larger than the cell diameter, local ridge-edge cues and ridge continuity remain sufficient to bias adhesion, spreading, and mechanotransduction at the population level. Consistently, quantitative analysis of F-actin immunofluorescence images showed more pronounced BMSC alignment on the 220 μm and 250 μm OCS groups than on the 70 μm TPMS control, as reflected by a narrower long-axis angle distribution and a higher alignment order parameter (Fig. S5). Its geometric order more closely resembles the anisotropic microenvironment represented by native lamellar bone, facilitating directional cell spreading and filopodia extension [47,48]. Moreover, the OCS groups exhibited greater fluid uptake, blood retention, and protein adsorption, which may contribute to a more favorable interfacial environment for cell attachment and tissue ingrowth [49]. In addition, the ordered stripes mitigate anisotropy in the transmission of structural cues, enabling cells to receive more stable and consistent mechanical stimulation and thus more efficiently initiate osteogenesis-related responses [41]. Consistently, the OCS showed higher VCL expression, increased FAK phosphorylation, and more pronounced YAP nuclear translocation, in agreement with enhanced expression of osteogenic markers such as RUNX2, OSX, and OCN. Notably, although the designed macropore architecture was consistent among groups, changing Hs simultaneously modulated the as-fabricated groove-ridge-like textures, total porosity, microporosity/interconnectivity, fluid uptake, mechanical properties, and ion release. Therefore, the superior osteogenic outcome of the 220 μm group is interpreted as resulting from a more favorable balance among interfacial microtexture, structural integrity, transport-related properties, mechanical support, and released-species profiles, rather than from microtexture alone. Accordingly, the present study cannot quantitatively separate the respective contributions of ion release and pore-wall topography to the *in vivo* outcome. Together, these factors likely account for the superior cytocompatibility and osteogenesis-related performance of the 220 μm and 250 μm groups, which was further supported by the *in vivo* outcomes.

Fabrication of groove-ridge-like textures within biodegradable Zn alloy porous scaffolds still faces another challenge: as the material degrades, the guiding effects of the surface textures on cells may gradually diminish. To address this issue, the present study further investigated how the groove-ridge-like textures influence osteogenesis *in vivo*. YAP-mediated mechanotransduction not only drives osteogenesis-related transcriptional programs but may also couple with epigenetic regulation. Previous studies have shown that YAP can influence the expression of histone modification-related enzymes through interactions with transcription factors and epigenetic regulators [50,51], and epigenetic regulation is a major route by which cells integrate microenvironmental cues and stabilize phenotypic states [52,53]. In this study, KDM5A downregulation and KDM6A upregulation were observed in the OCS groups both *in vitro* and *in vivo.* These consistent mRNA and protein-level changes indicate epigenetic regulator shifts; however, confirming chromatin remodeling would require direct readouts of histone marks (e.g., H3K4me3/H3K27me3) or chromatin accessibility assays. This may support a more persistent pro-osteogenic state at the nuclear level. *In vivo* analyses further showed mechanotransduction-associated signals and similar KDM5A/KDM6A expression trends during tissue ingrowth and correlated with improved regenerative outcomes; similar Hs-dependent trends observed in Ti scaffolds support a contribution of scaffold structural design in the absence of Zn^2+^/Li^+^ release, although the individual contributions of pore-wall texture, porosity, interconnectivity, and mechanics remain coupled. Overall, the OCS shifts the scaffold from passive structural support toward an active conditioning interface that enhances VCL-FAK-YAP mechanotransduction, osteogenic differentiation, and bone regeneration, accompanied by changes in KDM5A and KDM6A expression. Although major-organ H&E staining showed no evident off-target tissue damage, comprehensive systemic biosafety evaluation remains necessary. Future work will quantify time-resolved Zn^2+^/Li^+^ release, serum Zn^2+^/Li^+^ levels, pH evolution, mass loss, body weight changes, and hematological and biochemical indices to directly link scaffold degradation with osteogenic and systemic safety outcomes. Future studies will require experimental strategies that independently control topography, porosity, degradation, and released-species profiles to more rigorously define the contribution of each scaffold characteristic [15,54].

## 5 Conclusion

In summary, we fabricated a functionalized “Osteogenic Conditioning Scaffold” (OCS) with customized porous structure and delicately designed surface texture to tackle the unmet clinical challenge of large-sized bone defect repair. Through the combination of the selection of biodegradable Zn-0.4Li alloy and the precise modulation of L-PBF scanning parameters, we established a robust approach to achieve refined material-structure engineered OCS, thereby significantly boosting osseointegration efficacy and bone regenerative potential.

Inspired by field-ridge groove-ridge topography, the scaffold forms “orientation-controlled groove-ridge-like textures” inside the TPMS porous structure, which enhances interfacial wettability and can effectively promote cell adhesion. Without adding exogenous growth factors, the scaffold itself can enhance the adhesion and spreading of BMSCs, activate the cellular VCL-FAK-YAP mechanotransduction pathway, and be accompanied by changes in the expression of epigenetic regulators (downregulation of KDM5A and upregulation of KDM6A) to solidify the osteogenic phenotype, collectively enhancing *in vitro* osteogenesis.

In large-sized bone defects, conventional porous scaffolds often show limited marginal osseointegration and insufficient bone ingrowth toward the defect core, resulting in delayed and incomplete defect filling. By contrast, OCS improves both interface integration at the defect margins and bone ingrowth within the defect core by favorably releasing Zn^2+^ and Li^+^ ions and promoting tissue infiltration into its multilevel pore network. The *in vitro* mechanotransduction signals and epigenetic regulatory mechanisms were validated *in vivo*, providing comprehensive support for large-sized bone repair. Importantly, the OCS scaffold forms a complete “interface anchoring-internal ingrowth-central repair” regulatory system, addressing the core challenge of unstable interfaces and insufficient central regeneration in large-sized bone defect repair.

Overall, the OCS scaffold, with its unique structural design, robust manufacturing and bone regeneration promotion advantages, provides a new and efficient solution for large-sized bone defect repair, and has important clinical transformation value and application prospects.

## Data and materials availability

The data supporting the findings of this study are available from the corresponding author upon reasonable request.

## Ethics approval and consent to participate

All animal procedures were approved by the Animal Ethical Committee of Beijing Stomatological Hospital, Capital Medical University (Approval No. KQYY-202507-002), and were conducted in accordance with institutional guidelines.

## CRediT authorship contribution statement

**Xuanhe Feng:** Conceptualization, Data curation, Methodology, Project administration, Writing – original draft, Writing – review & editing. **Dongxu Xie:** Funding acquisition, Investigation, Supervision, Validation, Writing – review & editing. **Qiunan Zhou:** Data curation, Formal analysis, Validation, Writing – original draft. **Wanzhen Lei:** Data curation, Formal analysis, Validation. **Yilin He:** Investigation, Methodology. **Chen Zhang:** Formal analysis. **Siyuan Ouyang:** Methodology. **Lei Hu:** Project administration, Writing – review & editing. **Peng Wen:** Funding acquisition, Methodology, Writing – review & editing. **Luyuan Jin:** Funding acquisition, Project administration, Writing – review & editing.

## Declaration of competing interest

The authors declare the following financial interests/personal relationships which may be considered as potential competing interests:

Peng Wen is an editorial board member for Bioactive Materials and was not involved in the editorial review or the decision to publish this article. All authors declare that there are no competing interests.
