# Supplementary material for: Field-ridge structured bioactive Zn-based scaffold promotes bone regeneration via cellular mechanotransduction
Source: Bioact Mater. 2026 Aug 5;67:213–29. doi: 10.1016/j.bioactmat.2026.07.048 (PMC13471069; doi:10.1016/j.bioactmat.2026.07.048)
Supplement: Supplementary file 1 [file mmc1.docx]

**Supplementary Table S1. Primer sequences used for RT–qPCR analysis**

| Gene | Species | Forward (5′→3′) | Reverse (5′→3′) |
| --- | --- | --- | --- |
| β-actin | Rat | CACCCGCGAGTACAACCTT | TCATCCATGGCGAACTGGTG |
| Runx2 | Rat | GGCCACTTACCACAGAGCTA | AGCTTCTGTCTGTGCCTTCT |
| Col1a1 | Rat | CTGACGCATGGCCAAGAAGAC | TGACTTCTGCGTCTGGTGATACATA |
| Sp7 | Rat | GCCACCCATTGCCAGTAATC | ATAGTGAGCTTCTTCCTGGGG |
| Bmp2 | Rat | AGGCACCCTTTGTATGTGGA | TATGGCATGGTTGGTGGAGT |
| Bglap | Rat | CCCAATTGTGACGAGCTAGC | CCATAGATGCGCTTGTAGGC |
| Bsp | Rat | CTGAAGCCTGACCCATCTCA | TCGTCGTCATCATCGTCCAT |
| Kdm5a | Rat | ACAGCACGTTCCGCAACTAT | CAGGTGACTAGGGTCTTGACG |
| Kdm6a | Rat | TGACCAATTTACATTAGCTCCTCC | CTCCACACTTGGAAGGTTGC |
