# Supplementary material for: Field-ridge structured bioactive Zn-based scaffold promotes bone regeneration via cellular mechanotransduction
Source: Bioact Mater. 2026 Aug 5;67:213–29. doi: 10.1016/j.bioactmat.2026.07.048 (PMC13471069; doi:10.1016/j.bioactmat.2026.07.048)
Supplement: Supplementary file 2 [file mmc2.docx]

**Supplementary Table S2. Antibodies used for Western blot analysis.**

| Target | Host | Company | Catalog No. | Dilution |
| --- | --- | --- | --- | --- |
| p-YAP (Ser127) | Rabbit | Cell Signaling Technology | D9W2I | 1:1000 |
| YAP | Rabbit | Cell Signaling Technology | 14074 | 1:1000 |
| Vinculin (VCL) | Mouse | Santa Cruz Biotechnology | 73614 | 1:1000 |
| Osteocalcin (OCN) | Rabbit | Abclonal | A20800 | 1:1000 |
| RUNX2 | Rabbit | Bioss | bs1134R | 1:1000 |
| Osterix (OSX) | Rabbit | Bioss | bs1110R | 1:1000 |
| FAK | Rabbit | Abcam | ab40794 | 1:1000 |
| p-FAK (Tyr397) | Rabbit | Abcam | ab81298 | 1:1000 |
| GAPDH | Mouse | Proteintech | 60004-1-Ig | 1:50000 |
| HRP-Goat Anti-Rabbit Recombinant Secondary Antibody | Goat | Proteintech | RGAR001 | 1:10000 |
| HRP-Goat Anti-Mouse Recombinant Secondary Antibody | Goat | Proteintech | RGAM001 | 1:10000 |
