# Supplementary material for: Field-ridge structured bioactive Zn-based scaffold promotes bone regeneration via cellular mechanotransduction
Source: Bioact Mater. 2026 Aug 5;67:213–29. doi: 10.1016/j.bioactmat.2026.07.048 (PMC13471069; doi:10.1016/j.bioactmat.2026.07.048)
Supplement: Supplementary file 3 [file mmc3.docx]

**Supplementary Table S3. Antibodies used for immunofluorescence staining.**

| Target | Host | Company | Catalog No. | Dilution |
| --- | --- | --- | --- | --- |
| p-YAP (Ser127) | Rabbit | Abiowell | AWA10349 | 1:200 |
| YAP | Rabbit | Abiowell | AWA10217 | 1:200 |
| Osterix (OSX) | Rabbit | Abiowell | AWA13682 | 1:200 |
| KDM5A | Rabbit | Abiowell | AWA14189 | 1:200 |
| KDM6A | Rabbit | Abclonal | A27546 | 1:200 |
| Anti-rabbit IgG (H+L), Fab2 Alexa Fluor® 488 Conjugate | Goat | Cell Signaling Technology | 4412S | 1:1000 |
| Anti-mouse IgG (H+L), Fab2 Alexa Fluor® 594 Conjugate | Goat | Cell Signaling Technology | 8890S | 1:1000 |
