# Supplementary material for: Field-ridge structured bioactive Zn-based scaffold promotes bone regeneration via cellular mechanotransduction
Source: Bioact Mater. 2026 Aug 5;67:213–29. doi: 10.1016/j.bioactmat.2026.07.048 (PMC13471069; doi:10.1016/j.bioactmat.2026.07.048)
Supplement: Supplementary file 4 [file mmc4.docx]

**SUPPLEMENTTARY DATA**

**Fig. S1** Cytocompatibility and mechanical performance of Zn-0.4Li scaffolds fabricated with different Hs. (A, B) Representative live/dead staining images and corresponding quantitative analysis for Zn-0.4Li scaffolds with different structures; (C) Young’s modulus; (D) compressive strength. Data are presented as mean ± SD.

**Fig. S2** Healing outcomes of rabbit calvarial defects treated with Ti scaffolds. (A) Representative 3D micro-CT reconstructions at 1 month post-implantation; (B) bone volume fraction (BV/TV) of newly formed bone around the scaffolds at 1 month post-implantation; (C) BV/TV of newly formed bone within the defect region at 1 month post-implantation; (D) representative 3D micro-CT reconstructions at 2 months post-implantation; (E) BV/TV of newly formed bone around the scaffolds at 2 months post-implantation; (F) BV/TV of newly formed bone within the defect region at 2 months post-implantation; (G) methylene blue/acid fuchsin (MB/AF) staining at 1 month post-implantation; (H) H&E staining at 1 month post-implantation. Data are presented as mean ± SD.

**Fig. S3** Histological evaluation of rabbit calvarial defect healing with Ti scaffolds. (A) Sequential fluorochrome labeling with alizarin red and calcein; (B-F) immunofluorescence staining of regenerated tissue within the defect region; (G) quantitative analysis of fluorochrome labeling; (H-L) semiquantitative analysis of immunofluorescence staining. Data are presented as mean ± SD.

**Fig. S4** *Kdm5a* and *Kdm6a* expression is not altered under extract-only conditions. (A,B) RT-qPCR analysis of *Kdm5a* (A) and *Kdm6a* (B) mRNA levels in BMSCs cultured under extract-only conditions. Data are presented as mean ± SD.

**Fig. S5** Quantification of BMSC alignment on scaffolds with different pore-wall microtextures. (A-C) Histograms showing the distribution of cell long-axis alignment angles (θ, 0–90°) for the 70 μm (A), 220 μm (B), and 250 μm (C) groups; (D) Alignment order parameter calculated as S = ⟨cos(2θ)⟩, where larger S indicates stronger alignment.

**Fig. S6** Protein adsorption on scaffolds. (A, B) Quantification of adsorbed serum protein on the 70 μm, 220 μm, and 250 μm scaffolds after incubation for 4 h (A) and 24 h (B). Adsorbed proteins were eluted and quantified using a Bradford assay, and the protein amount was normalized to the exposed scaffold surface area. The 220 μm and 250 μm OCS groups showed higher protein adsorption than the 70 μm TPMS control. Data are presented as mean ± SD.

**Fig. S7** Surface morphology and topographic characterization of groove-ridge-like textures on scaffold pore-wall surfaces. (A-C) Confocal surface topography maps and representative line profiles of the 70 μm (A), 220 μm (B), and 250 μm (C) groups; (D) SEM images of the surface morphology; (E) Micro-CT reconstruction of the surface morphology; (F) Quantification of arithmetic mean surface roughness (Sa). Data are presented as mean ± SD.

**Fig. S8** Zn²⁺ and Li⁺ concentrations in scaffold extracts. (A, B) Quantification of Zn²⁺ (A) and Li⁺ (B) concentrations in extracts from the 70 μm, 220 μm, and 250 μm scaffolds under the extract conditions used for *in vitro* assays. Data are presented as mean ± SD.

**Fig. S9** Histological evaluation of major organs at 8 weeks after Zn-0.4Li scaffold implantation. (A, B) Representative H&E staining images of the lung, heart, liver, spleen, and kidney from the blank (A) and Zn-0.4Li scaffold (B) groups.
